# Supplementary material for: Structural mechanism of insulin receptor activation by a dimeric aptamer agonist
Source: Exp Mol Med. 2025 Jul 2;57(7):1506–18. doi: 10.1038/s12276-025-01494-1 (PMC12322039; doi:10.1038/s12276-025-01494-1)
Supplement: Supplementary file 1 — Supplementary Information [file 12276_2025_1494_MOESM1_ESM.pdf]

Supplementary Information

Structural comparison of $IR_{pseudo\text{-}gamma}$ with $IR_{pseudo\text{-}arrowhead}$ and $IR_{arrowhead}$

To investigate the structural changes from $IR_{pseudo\text{-}arrowhead}$ to $IR_{pseudo\text{-}gamma}$, we aligned the stalks of protomer A (Supplementary Fig. 7a). In $IR_{pseudo\text{-}gamma}$, the aptamer-free head (L1-CR domain) of protomer A is elevated by 10.2º compared to the aptamer-bound protomer A of $IR_{pseudo\text{-}arrowhead}$ (Q177-L459-V604; L1-L2-FnIII2). Meanwhile, the aptamer-bound protomer B in $IR_{pseudo\text{-}gamma}$ is downshifted by 7.2º relative to protomer B in $IR_{pseudo\text{-}arrowhead}$ (Supplementary Fig. 7b). The L1 domain of protomer A in $IR_{pseudo\text{-}gamma}$ has shifted by 29.5 Å, while in protomer B, it has shifted by 16.4 Å.

Alignment of the L2 domains between $IR_{pseudo\text{-}gamma}$ and $IR_{pseudo\text{-}arrowhead}$ revealed that protomer A of $IR_{pseudo\text{-}gamma}$ adopts a more compact form (Q177-E204-L459; L1-CR-L2, 52º) compared to protomer A of $IR_{pseudo\text{-}arrowhead}$ (78º). In contrast, protomer B of $IR_{pseudo\text{-}gamma}$ maintains a similar conformation to protomer A of $IR_{pseudo\text{-}arrowhead}$ (86º vs. 83º) (Supplementary Fig. 7c, d). The distance between L1 domains in protomer A and B of $IR_{pseudo\text{-}gamma}$ is 43.3 Å and 6.7 Å, respectively. Overall, protomer A undergoes significant changes at two hinge regions (CR-L2 and L2-FnIII1), while protomer B shows relatively smaller changes, particularly in the L2-FnIII1 hinge.

When aligning the stalk of $IR_{pseudo\text{-}gamma}$ with that of $IR_{arrowhead}$, one head shifts upward while the other shifts downward at the L2-FnIII1 hinge (Supplementary Fig. 7e, f). The ligand-free protomer A is uplifted by 19.3º and 15º at Q177-L459-V604 (L1-L2-FnIII2) and E204-L459-V604 (CR-L2-FnIII2), respectively, while the aptamer-bound protomer B is downshifted by 1.1º and 10.6º. The L1 domain of protomer A shifts by 48 Å, while in protomer B, it shifts by 26 Å.

Upon aligning the L2 domains, the aptamer-free protomer A of $IR_{pseudo\text{-}gamma}$ forms a more compact structure (angle reduced from 82º to 52º), while the aptamer-bound protomer B retains a nearly identical conformation (angle shift from 82º to 86º) (Supplementary Fig. 7g, h). The distance between L1-L1' or CR-CR' is much smaller for protomer B (5 Å) compared to protomer A (60 Å and 44 Å, respectively). Thus,

protomer A experiences substantial shifts at two hinges, while the L1-FnIII1 hinge of aptamer-bound protomer B undergoes only minor adjustments.

Supplementary Figure Legends

Supplementary Figure 1. Effects of the dose and duration of A62M and A62D-8T on IR phosphorylation and downstream signaling

(a) Comparison of dose-dependent phosphorylation of IR and downstream signaling by A62M or A62D-8T. Rat-1/hIR cells were incubated with varying concentrations of A62M or A62D for 1 h. (b) The relative band intensities, compared to 100 nM insulin, are shown for pIR Y1150, pIR Y1150/Y1151, pAKT T308, pAKT S473, and pERK T202/Y204. Quantified values for the band intensities are shown in Fig 2.

Supplementary Figure 2. Purification of the IR + A62D complex, analysis of the quality of the cryo-EM map (a) Size-exclusion chromatography profile and SDS-PAGE result. (b) Representative cryo-EM micrograph of the IR + A62D complex. **c-e,** 2D class averages of (c) $IR_{arrowhead}$, (d) $IR_{pseudo\text{-}arrowhead}$ and (e) $IR_{pseudo\text{-}gamma}$. **f-n**, Fourier shell correlation curves (**f-h**), Angular distributions (**i-k**) and Local resolution of the map (**l-n**) of (**f, i, l**) $IR_{arrowhead}$, (**g, j, m**) $IR_{pseudo\text{-}arrowhead}$ and (**h, k, n**) $IR_{pseudo\text{-}gamma}$.

Supplementary Figure 3. Workflow of cryo-EM processing for the $IR_{arrowhead}$. Flow chart of data processing for the $IR_{arrowhead}$.

Supplementary Figure 4. Workflow of cryo-EM processing for the $IR_{pseudo\text{-}arrowhead}$

Supplementary Figure 5. Workflow of cryo-EM processing for the $IR_{pseudo\text{-}gamma}$

Supplementary Figure 6. Close-up view of the aptamer binding site in IR_{pseudo-}

$_{arrowhead}$ (a) A cartoon representation of the rigid-body rotation of the IR_{apo} (gray) to the $IR_{arrowhead}$ (orange). Only one protomer is shown for clarity. (b) Rigid-body rotation in a is shown in a 90°-rotated view. (c) A close up view of the cryo-EM density for the A62D in the $IR_{pseudo\text{-}arrowhead}$. Only one A62M module of the A62D can be modeled into the density. (d) Cryo-EM density for the A62D in $IR_{pseudo\text{-}arrowhead}$. (e) Interaction of A62M with the L1 and FnIII-1 domains at site l and site u in $IR_{pseudo\text{-}arrowhead}$. (f) Close-up view of the site-l interface, (g) Close-up view of the site-u interface. (h) Superimposed structures of the $IR_{arrowhead}$ and $IR_{pseudo\text{-}arrowhead}$ by aligning protomer A. Comparison of the B protomers between $IR_{arrowhead}$ and $IR_{pseudo\text{-}arrowhead}$ illustrates the rigid-body rotation of protomer B. (i) Aligned structures of $IR_{arrowhead}$ (white) and $IR_{pseudo\text{-}arrowhead}$ (yellow, blue). Distance between the membrane-proximal ends of the FnIII-3 stalks (D907-D907') is shown for both IRs.

Supplementary Figure 7. Structural comparison of $IR_{pseudo\text{-}gamma}$ with $IR_{pseudo\text{-}arrowhead}$ and $IR_{arrowhead}$

(a, b) Superimposed structures of protomer A (a) and protomer B (b) in $IR_{pseudo\text{-}gamma}$ (orange) with the respective protomers in $IR_{pseudo\text{-}arrowhead}$ (blue), aligned by stalks. (c, d) Superimposed structures of protomer A (c) and protomer B (d) in $IR_{pseudo\text{-}gamma}$ (orange) with the respective protomers in $IR_{pseudo\text{-}arrowhead}$ (blue) aligned by the L2 domains. Protomers of the $IR_{pseudo\text{-}gamma}$ and $IR_{pseudo\text{-}arrowhead}$ are shown in orange and blue, respectively. (e, f) Superimposed structures of protomer A (e) and protomer B (f) in $IR_{pseudo\text{-}gamma}$ (orange) with the respective protomers in $IR_{arrowhead}$ (blue), aligned by stalks. (g, h) Superimposed structures of protomer A (g) and protomer B (h) in $IR_{pseudo\text{-}gamma}$ with the respective protomers in $IR_{arrowhead}$, aligned by the L2 domains. Protomers of the $IR_{pseudo\text{-}gamma}$ and $IR_{arrowhead}$ are shown in orange and blue, respectively.

Supplementary Figure 8. A62D aptamer induces oligomerization of IR

(a) Quantification of IR signal intensities in puncta. Data are presented as mean±SD (standard deviation) to illustrate the distribution of signal intensities. The number of puncta analyzed is as follows: A62M, 100nM: 42; A62M, 200nM: 274; A62M, 400nM:

173; A62D, 50nM: 508; A62D, 100nM: 345; A62D, 200nM: 396.

(b) Quantification of IR signal intensities in puncta localized in the plasma membrane. The plasma membrane was defined as the F-actin-stained region, and IR puncta within this region were analyzed. Data are presented as mean±SEM (standard error of the mean). The number of puncta analyzed is as follows: A62M, 100nM: 19; A62M, 200nM: 106; A62M, 400nM: 66; A62D, 50nM: 179; A62D, 100nM: 106; A62D, 200nM: 175.

(c) Quantification of IR signal intensities in puncta in the cytoplasm. The cytoplasmic region was defined as the area excluding both the F-actin- and DAPI-stained regions. Data are presented as mean±SEM. The number of puncta analyzed is as follows: A62M, 100nM; 24, A62M, 200nM: 132; A62M, 400nM: 85; A62D, 50nM: 549; A62D, 100nM: 303; A62D, 200nM: 304.

(d) Quantification of IR signal intensities in puncta in the nucleus. The nuclear region was defined as the DAPI-stained area. Data are presented as mean±SEM. The number of puncta analyzed is as follows: A62M, 200nM: 38; A62M, 400nM: 3; A62D, 50nM: 94; A62D, 100nM: 52; A62D, 200nM: 60.

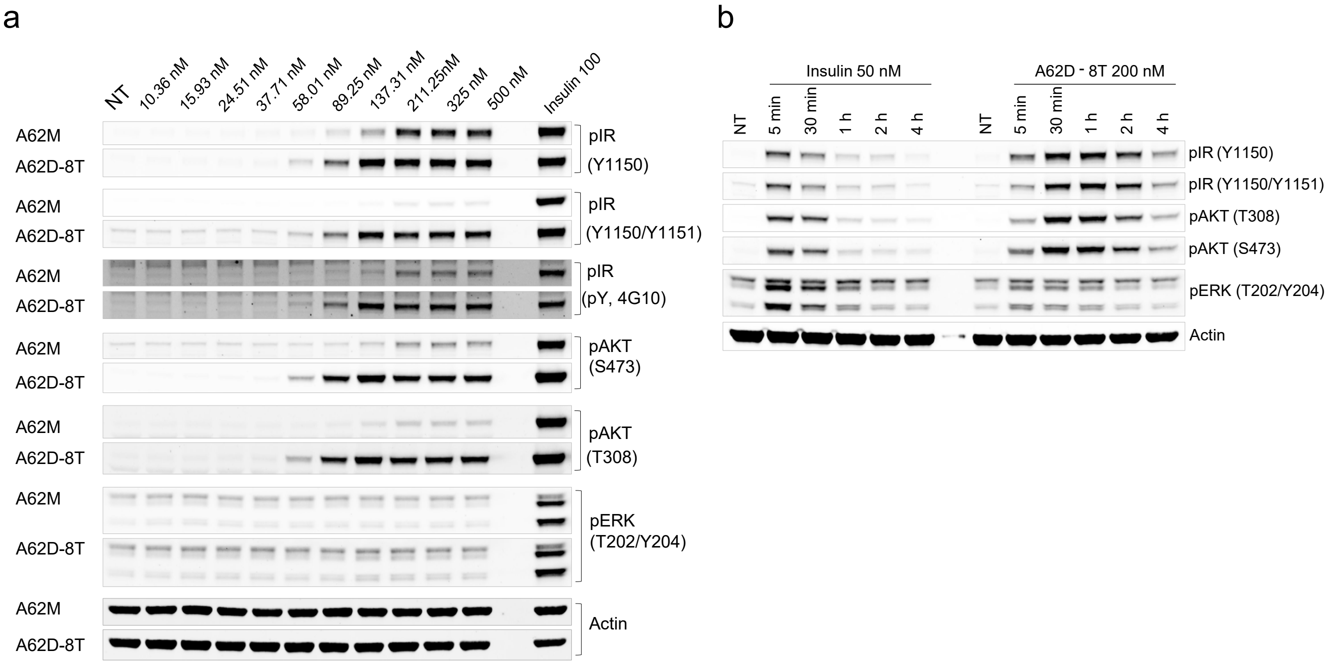

Supplementary Figure 1

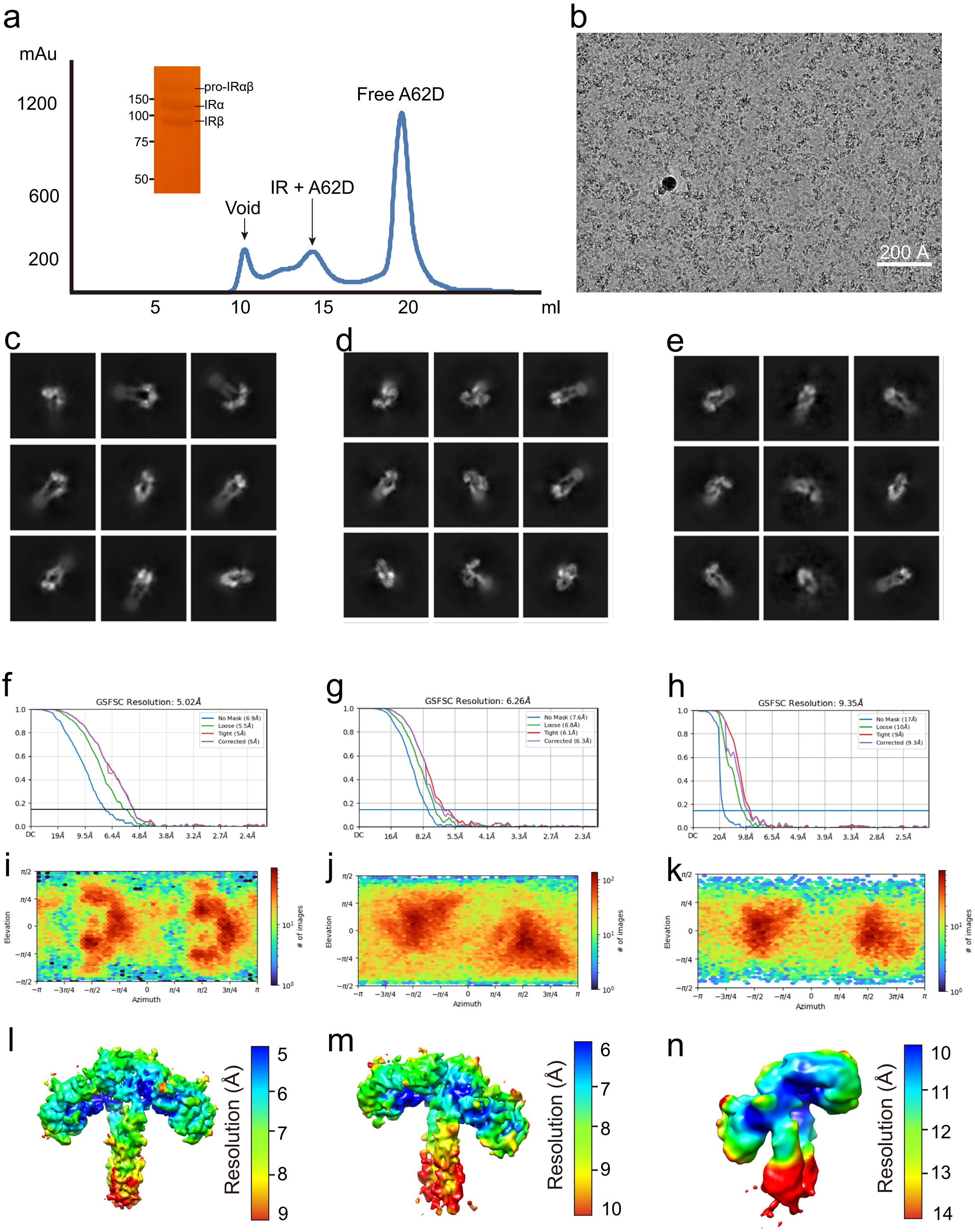

Supplementary Figure 2

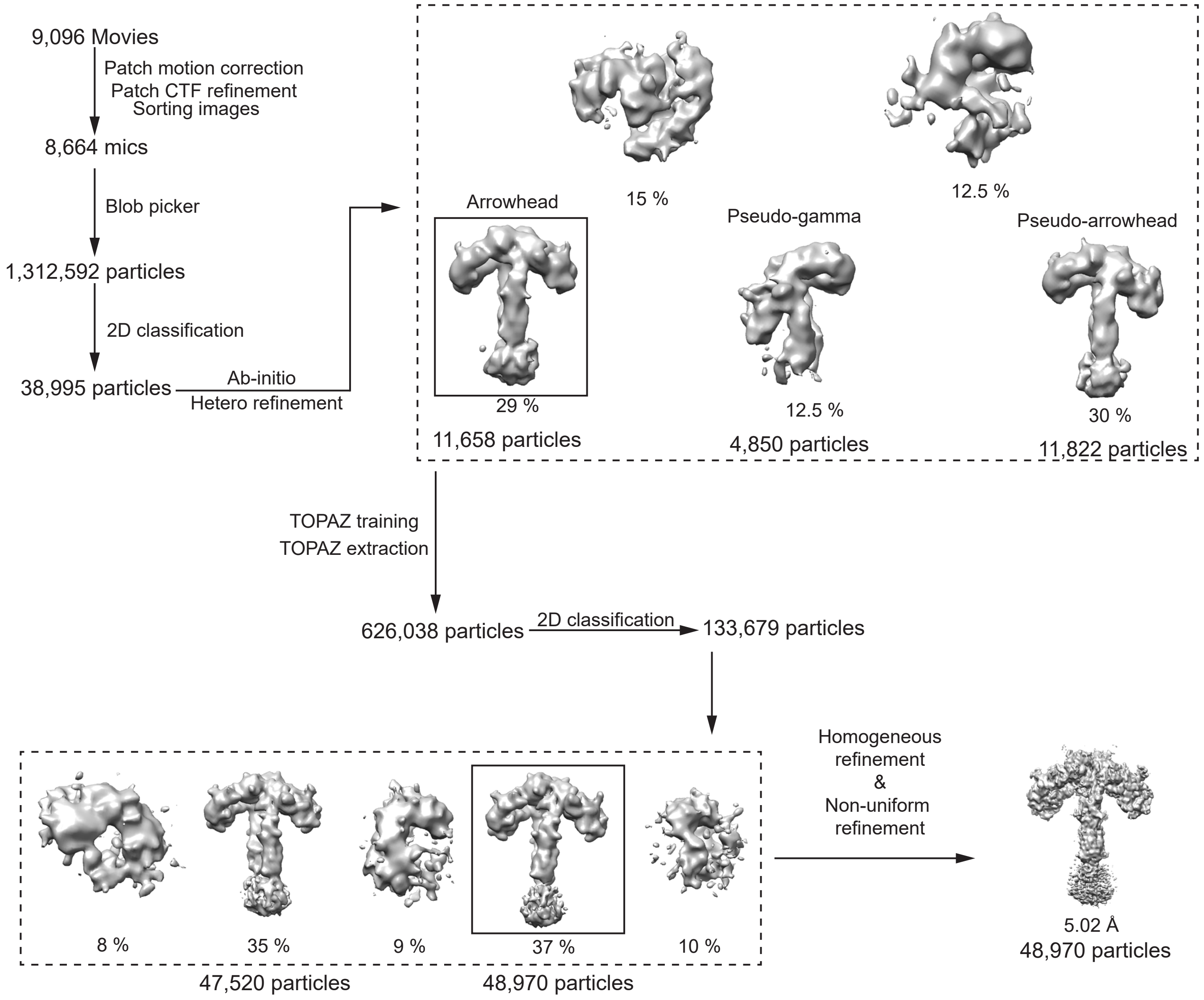

Supplementary Figure 3

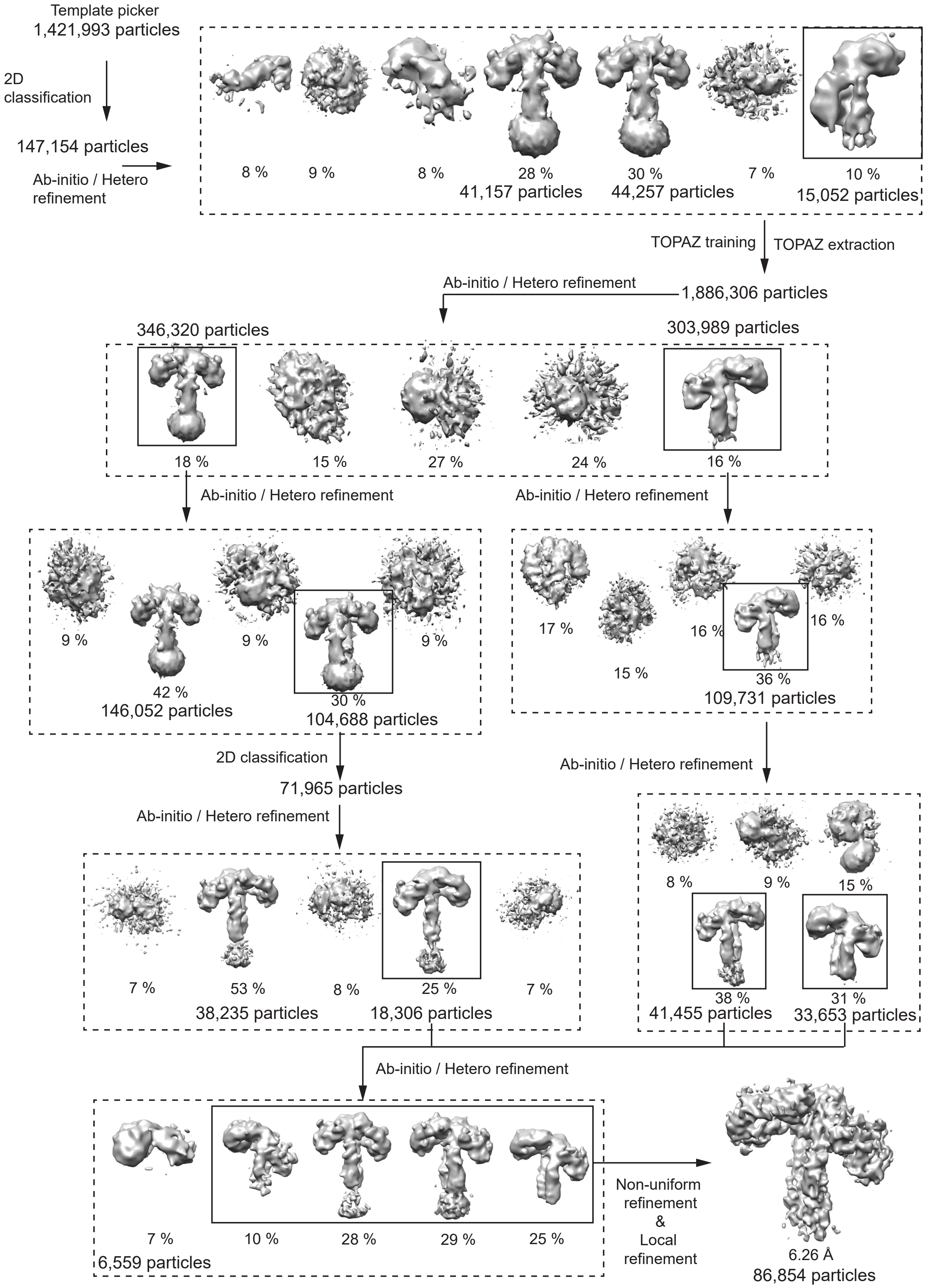

Supplementary Figure 4

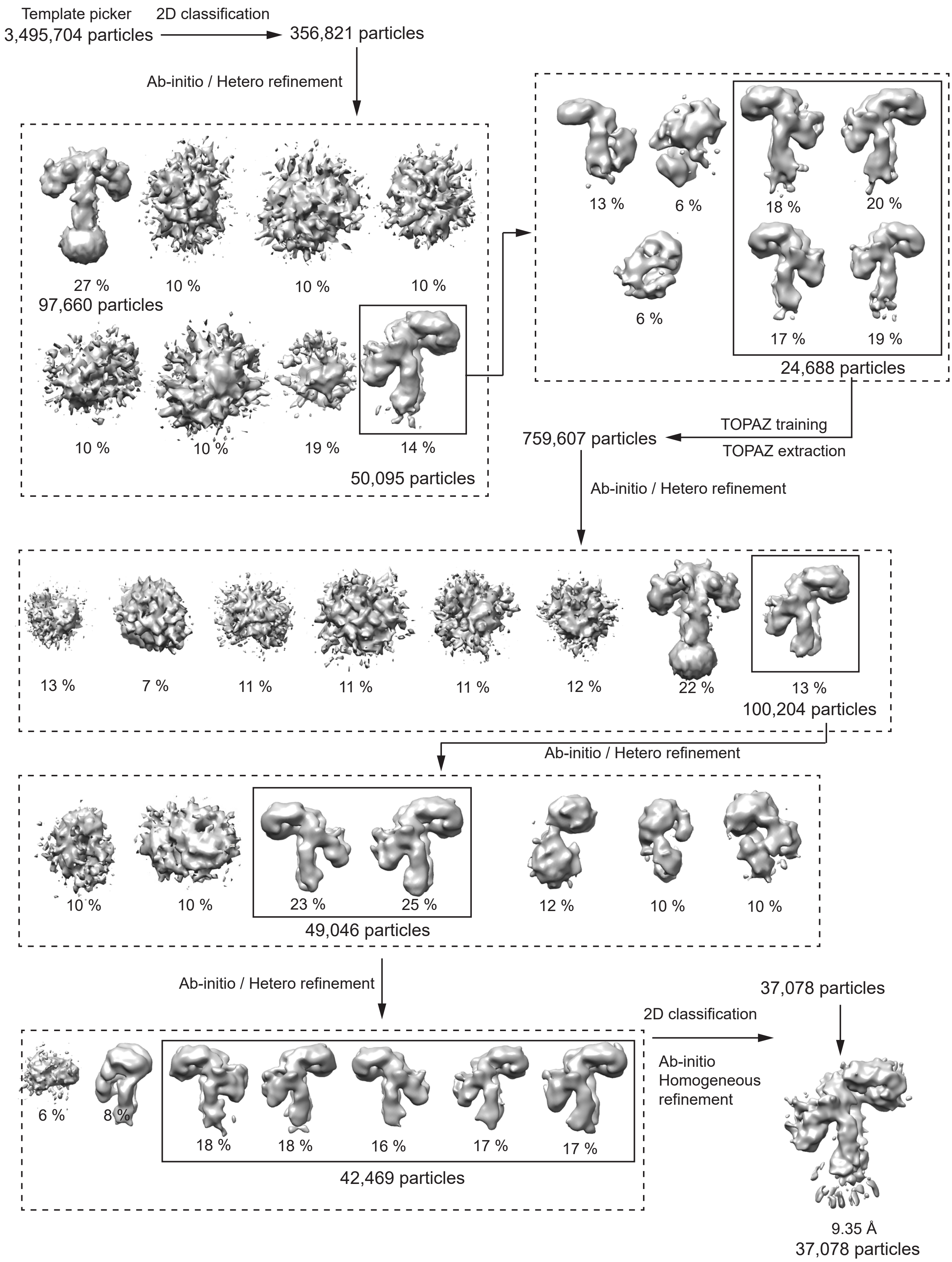

Supplementary Figure 5

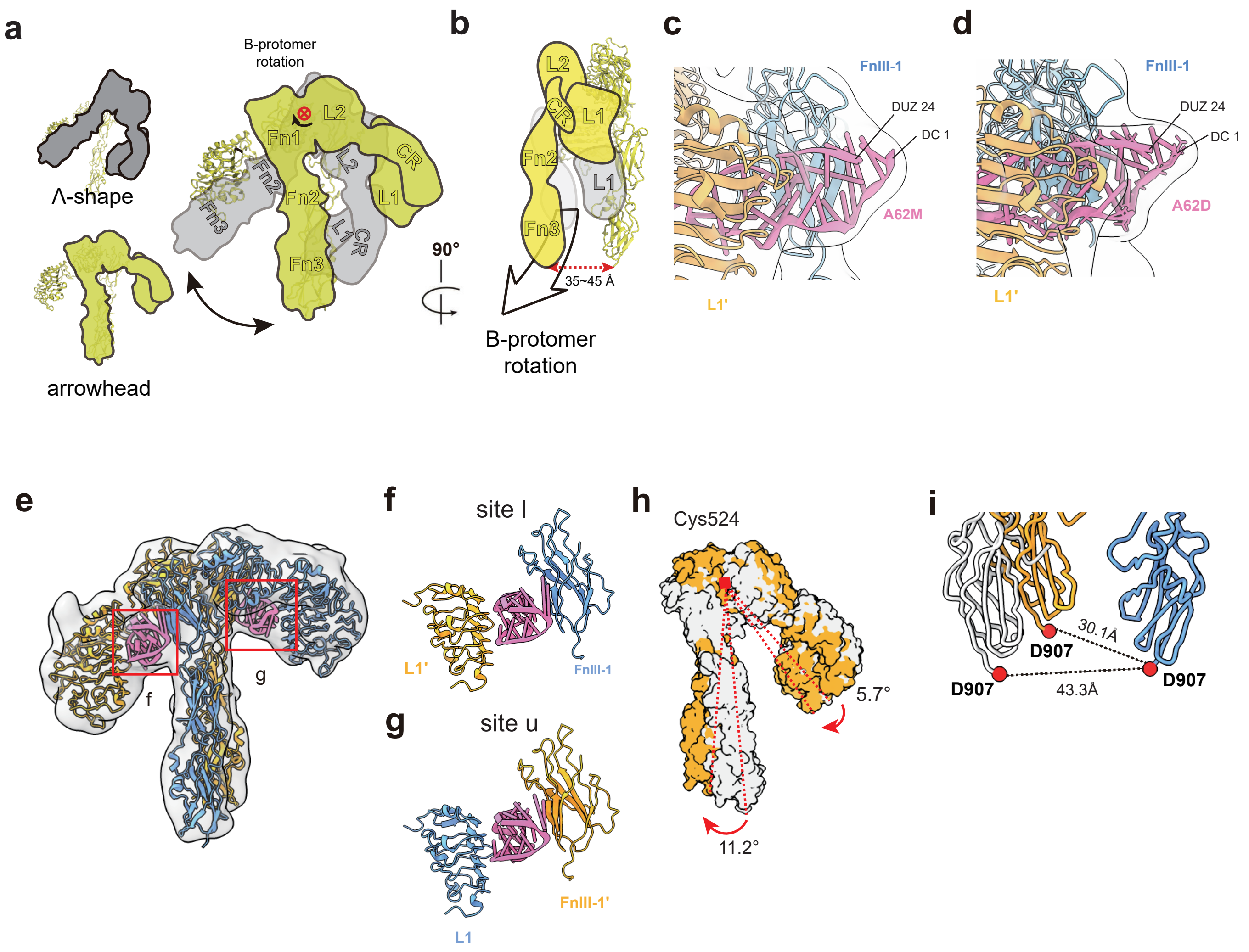

Supplementary Figure 6

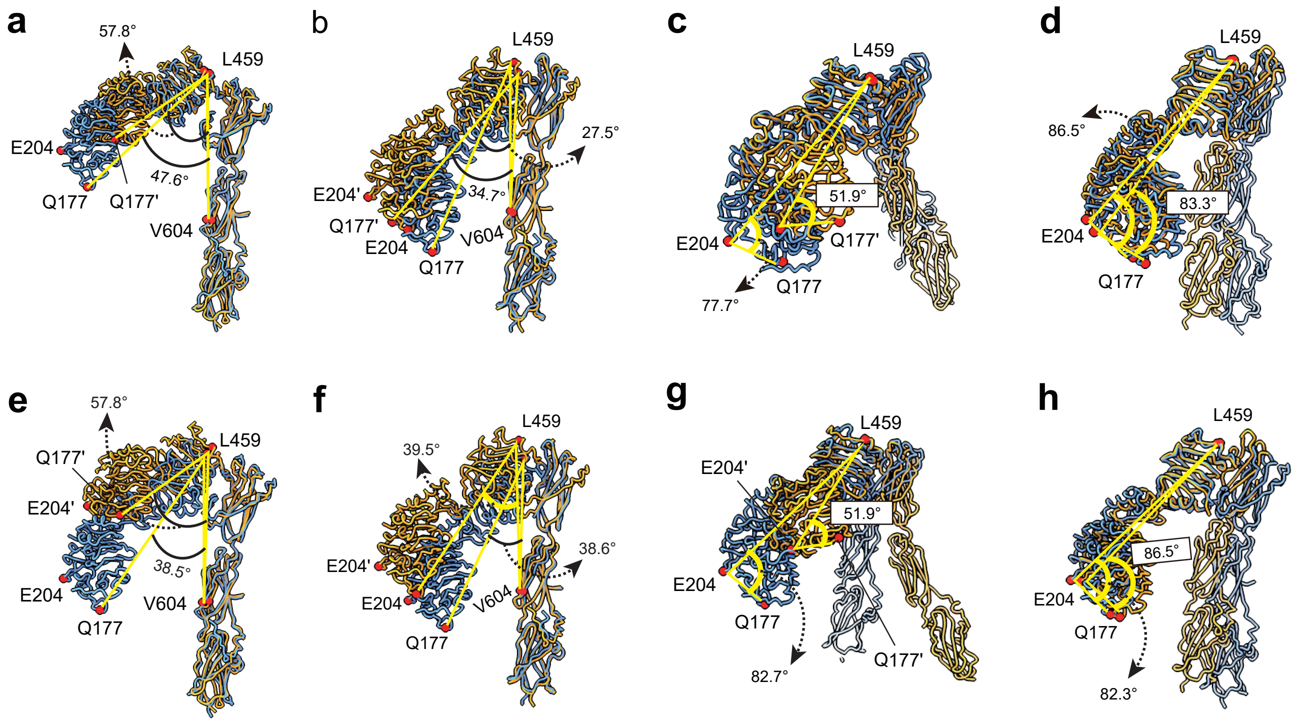

Supplementary Figure 7

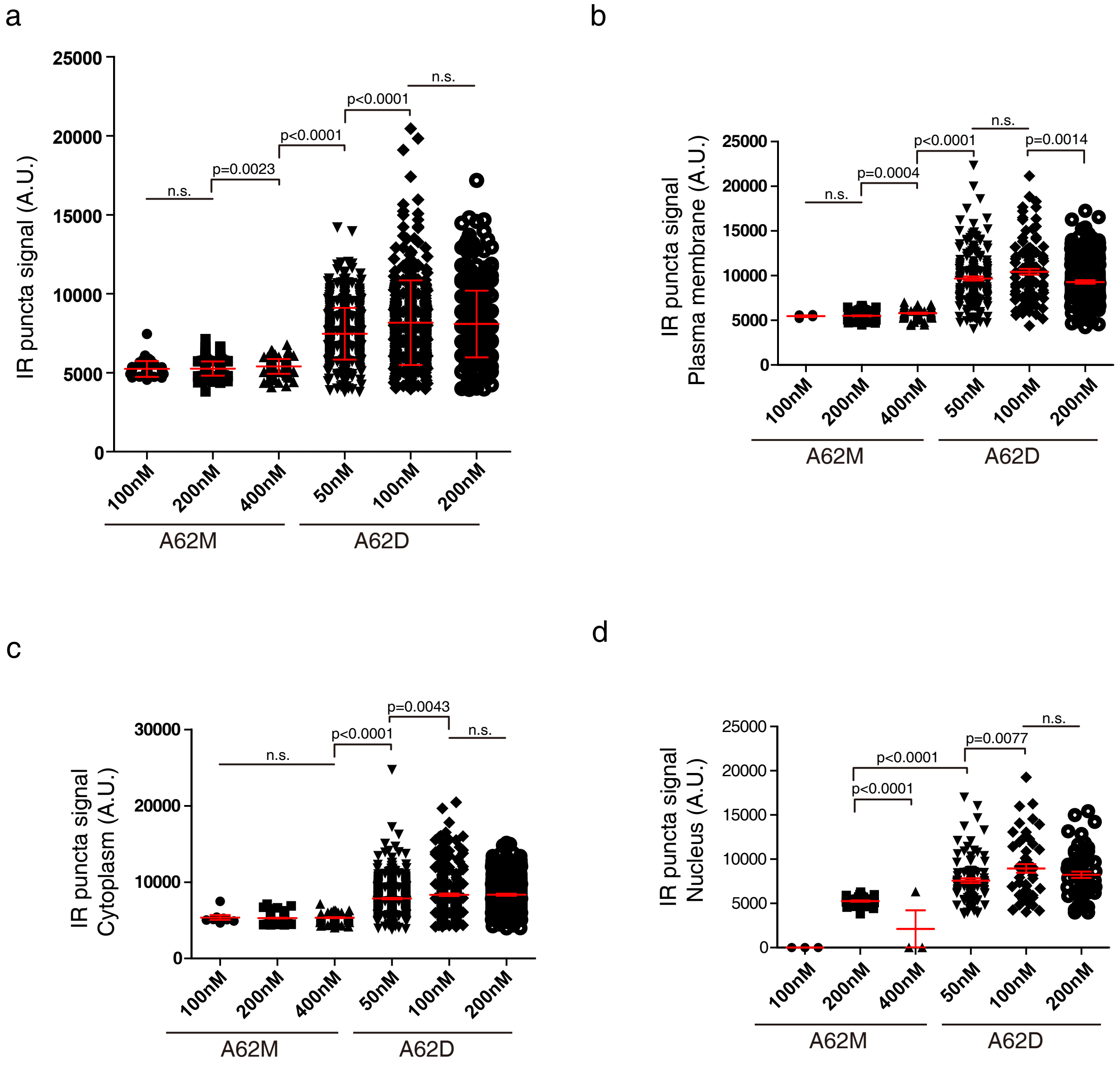

Supplementary Figure 8

Supplementary Table 1 | Cryo-EM data collection, refinement and validation statistics.

	[a]$IR_{arrowhead}$ (EMD-61490) (PDB 9JHS)	[a]$IR_{pseudo\text{-}arrowhead}$ (EMD-61431) (PDB 9JF9)	[a]$IR_{pseudo\text{-}gamma}$ (EMD-61432) (PDB 9JFD)
Magnification	79,000	79,000	79,000
Voltage (kV)	300	300	300
Electron exposure ($e^{-}/Å^2$)	50	50	50
Defocus range (μm)	-1.0 to -2.0	-1.0 to -2.0	-1.0 to -2.0
Pixel size (Å)	1.0902	1.0902	1.0902
Symmetry imposed	C1	C1	C1
Initial particle images (No.)	1,312,592	1,421,993	3,495,704
Final particle images (No.)	48,970	86,854	37,078
Map resolution (Å)	5.02	6.26	9.35
FSC threshold	0.143	0.143	0.143
Initial model (PDB code)	7YQ6	7YQ6	7YQ3/ 7YQ6
Model resolution (Å)	4.9	6.2	16.6
FSC threshold	0.143	0.143	0.143
Map sharpening *B* factor ($Å^2$)	-224.5	-469.5	-263.8
Model composition			
Non-hydrogen atoms	13,990	14,032	12,970
Protein residues	1,592	1,589	1,533
Nucleotide	48	48	24
B factors ($Å^2$)			
Protein	525.8	312.3	1009.26
Nucleotide	319.68	255.63	969.35
R.m.s. deviations			
Bond lengths (Å)	0.003	0.003	0.003
Bond angles (°)	0.879	0.874	0.862
Validation			
MolProbity score	2.29	2.32	2.29
Clashscore	17.7	21.47	21.76
Poor rotamers (%)	0	0	0.29
Ramachandran plot			
Favored (%)	90.25	91.72	92.83
Allowed (%)	9.75	8.28	7.03
Disallowed (%)	0	0	0.13

[a] Same data set was used
